# TUSC3, p53 and p21 genetic association with development of oral submucous fibrosis and oral squamous cell carcinoma among addictive tobacco chewers of Pakistan

**DOI:** 10.1186/s12903-024-04501-5

**Published:** 2024-07-11

**Authors:** Syed Aqib Ali Zaidi, Nadia Chughtai, Zubair Ahmed Abbassi, Jehan Alam, Tuba Shakil Malick, Asmat Salim, Saima Saleem

**Affiliations:** 1 The Karachi Institute of Biotechnology and Genetic Engineering (KIBGE), University of Karachi, Karachi, Pakistan; 2 Present Address: Shenzhen Key Laboratory of Anti-Aging and Regenerative Medicine, Shenzhen University Medical School, Shenzhen University, Shenzhen, 518060 China; 3 Karachi Medical and Dental Collage, Karachi, Pakistan; 4 Department of Dentistry, Jinnah Sindh Medical University (JSMU), Karachi, Pakistan; 5 Department of Dentistry, Jinnah Postgraduate Medical Centre (JPMC), Karachi, 75510 Pakistan; 6 Dr. Panjwani Center for Molecular Medicine and Drug Research (PCMD), International Center for Chemical and Biological Sciences (ICCBS), University of Karachi, Karachi, Pakistan

**Keywords:** Oral squamous cell carcinoma (OSCC), Oral Submucous Fibrosis (OSF), TUSC3, TP53, p21, Early diagnostic marker

## Abstract

**Background:**

This study delves into the intricate landscape of oral cancer, a global concern with a high incidence in Asian countries. We focus on oral squamous cell carcinoma (OSCC), primarily driven by the consumption of betel nut and its derivatives. OSCC often arises from premalignant lesions like oral submucous fibrosis (OSF). In Pakistan, OSCC is prevalent among men due to various addictive substances, including smokeless tobacco and chewing materials. Mutations in tumor suppressor genes, such as TP53 and p21, play crucial roles in this malignancy’s development. We also explore the involvement of TUSC3 gene deletion in OSCC and OSF.

**Methods:**

In this study we investigated demographics, TUSC3 gene expression, deletion analysis, and TP53 and p21 genetic alterations in OSCC and OSF patients (blood and tissue of 50 samples in each condition) who had tobacco derivates usage history. The association analysis was carried out mainly through PCR based genotyping.

**Results:**

The study’s patient cohort (OSCC and OSF) displayed a wide age range from 13 to 65 years (Mean = 32.96 years). Both conditions were more prevalent in males, with a male-female ratio of approximately 2.5:1. Chewing habits analysis revealed high frequencies of gutka use in both OSF and OSCC patients. TUSC3 expression analysis in OSCC cell lines indicated significant downregulation. Genotyping showed no TUSC3 deletion in OSF cases, but a deletion rate of over 22% in OSCC tissue samples. Analysis supported a significant association of TUSC3 deletion with OSCC development but not with OSF. Polymorphism in p53 exon 4 and p21 (rs1801270) were significantly associated with both OSCC and OSF, adding to their pathogenesis. Our findings further revealed a strong correlation between TUSC3 deletion and the excessive use of tobacco and related products, shedding light on the genetic underpinnings of OSCC development.

**Conclusions:**

Notably, our study provides a crucial insight into genetic aspects underlying OSCC and OSF in response of addictive consumption of areca nut, betel quid, and tobacco derivatives. A significant association between TUSC3 deletion and OSCC development, along with polymorphisms in TP53 and p21, underscores the importance of further research into the molecular mechanisms driving oral cancer progression for improved diagnosis and treatment outcomes.

**Supplementary Information:**

The online version contains supplementary material available at 10.1186/s12903-024-04501-5.

## Background

Oral cancer is sixteenth ranked cancer of the world. More than 60% of oral cancer cases are reported from Asian countries. According to GLOBOCON statistics of 2022 among 185 countries, the incidence of oral cancer is 2% with mortality of 1.9%, the occurrence was found quite frequent in males especially of Asian region [1]. Approximately, 90% of oral cancers are oral squamous cell carcinoma (OSCC) [2], the cancer of the squamous cells present in the mucosal lining of the oral cavity [3]. Epidemiology based studies suggest that the one primary cause of OSCC development is the chewing of betel nut or its derivative materials [4, 5]. It has been observed that OSCC originates from pre-cancerous lesions of oral cavity known as oral potentially malignant disorders [6]. Oral submucous fibrosis (OSF) is a premalignant condition of oral cancer which may develop due to excessive use of chewing materials [7]. In OSF, a fibrous condition is developed in squamous cells of the oral cavity as a result of excessive deposition of collagen. Up to a quarter of OSF cases have shown epithelial dysplasia and have about 5.6–9.13% tendency to malignant transformation [8].

In Pakistan, OSCC is the most common malignancy in men. As it is a multifactorial disease, most cases share a common history of addictive materials like tobacco smoking, smokeless tobacco, areca nut and betel quid, and other chewing materials [9]. Their long term use leads to various types of oral cancers [10]. Chewing materials are available with different names according to their preparations; these are *paan, chaliya, niswar, gutka and manpuri.* These materials can be homemade or available commercially. The preparation of these chewing materials diverges according to products. *Paan* is locally prepared with a combination of different flavors and ingredients. *Niswar* is a kind of dipping tobacco prepared by crushing the fresh tobacco leaves with wood ash and calcium oxide. *Gutka* is a form of crushed areca nuts coated in tobacco and sweet agents. *Manpuri* is a powdered form of areca nuts and tobacco with slaked lime [11].

Many studies reported that mutations of tumor suppressor genes are one of the main causes of conversion from a premalignant condition to malignancy. These alterations are a result of a cascade of biochemical and histopathological changes [12]. There are multiple genes reported whose genetic changes may induce such conversion, like TP53, p21, and Tumor Suppressor Candidate (TUSC3) genes. TP53 and p21 have a well-established role in OSCC origination [13–15]. TP53 is a tumor suppressor gene essential for preventing cancer formation and progression. It governs the cell cycle and promotes programmed cell death (apoptosis) in cells with damaged DNA. When cells encounter DNA damage, TP53 is activated. It can either temporarily halt the cell cycle to allow DNA repair or trigger cell death if the damage is irreparable. Mutations in TP53 are common genetic alterations in various cancers, including oral cancer. These mutations are closely linked to tumor development and advancement [16]. However, p21 is a cyclin-dependent kinase inhibitor (CDKI) protein that acts as a cell cycle regulator. It can inhibit the activity of cyclin-dependent kinases (CDKs), which play a role in cell cycle progression. p21 is also recognized as a downstream target of TP53. When TP53 is activated due to DNA damage or other stress signals, it can stimulate the expression of p21. p21 has a function in blocking the cell cycle at the G1 and G2 checkpoints, providing time for DNA repair and stopping the replication of damaged DNA. It is a vital mediator of TP53’s cancer-suppressing function [17].

However, TUSC3 gene, present at chromosome 8, shows its candidature in the development of different cancers such as ovarian, colorectal, pancreatic [18], prostate [19], esophageal and pharyngeal cancers [20]. Previously this gene was known for its functions in learning and memory and is an active member of magnesium ion regulatory system in nerve cells. It encodes a 348 amino acid protein with an N-terminal and 4 transmembrane domains which are present within the endoplasmic reticulum membrane. TUSC3 encoded protein is mainly responsible for the N-glycosylation and maturation of nascent proteins [21]. Deletion or suppression of TUSC3 is ultimately responsible for immature proteins which may create stressed condition for the cell, which leads to abnormal function and growth.

In the current study, we analyzed the association of TUSC3 gene deletion, TP53 genetic alterations and p21 point mutation (*rs1801270*) in the development of the disease in OSCC and OSF patients.

## Materials and methods

### Sample collection

A total of 150 subjects were recruited in the study; 50 confirmed diagnosed cases of OSF and OSCC each, and 50 healthy individuals. After an informed consent, a tissue of 5 mm was surgically collected from OSF and OSCC patients. Similarly, 5 ml of blood were phlebotomized from all participants of the study, in an acid citrate dextrose (ACD) containing vacutainers. Samples were stored at -80 °C till further procedures.

### DNA extraction and quantification

DNA from peripheral blood and tumor was isolated by standard phenol chloroform extraction protocol and digestion by proteinase K. Extracted DNA samples were dissolved in TE buffer as per the size of the pellets. Quantitative analyses of DNA were performed by Nanodrop analyzer (IMPLEN NanoPhotometer® P-Class, Germany). One µl of each sample was placed on the sample rod and the respective cap was used to measure 260/280 ratio. Those samples which gave optical density between 1.7 and 1.9 were considered pure.

### Cell culture

Expression of TUSC3 gene was carried out on HGF1(ATCC® CRL2014™) and CAL 27 (ATCC® CRL2095™) cell lines. The maintenance and culture of HGF1 and CAL 27 was done in Gibco® Dulbecco’s Modified Eagle Medium (DMEM) supplemented with 10% Fatal Bovine Serum (FBS), sodium pyruvate, penicillin, and streptomycin at 37 °C and in a humidified condition under 5% CO_2_ in the biosafety level II cell culture facility of PCMD, ICCBS, University of Karachi. Fresh medium was added after every third day and sub-culturing was carried out by trypsinization once cells were 70–80% confluent. Cell morphology and conditions were regularly monitored by phase contrast microscope (Eclipse TE 2000-S, Nikon, Japan).

### Immunofluorescence

For further validation of TUSC3 expression in both cell lines, immunostaining was carried out. First a well containing cells was washed twice by PBS followed by fixation in 4% paraformaldehyde for 15 min at room temperature. Again, cells were washed with PBS twice and 0.1% triton x-100 was added for 10 min followed by rinsing twice in PBS. Cells were incubated in 2% bovine serum for 30 min at room temperature followed by washing and incubation with primary antibody against TUSC3 (D-9): sc-390,566 (SANTA CRUZ BIOTECHNOLOGY, INC) in 1:500 dilution overnight at 4 °C. Antibody was aspirated and cells were washed twice with PBS, and then incubated with secondary antibody Alexa Flour goat anti-mouse 546 for 1 h at room temperature in dark. It was followed by PBS rinsing, twice and incubating the cells with 1 µg/ml DAPI. Cells were mounted and analyzed for immunofluorescence with fluorescent microscope (TE 2000-S, Nikon, Japan).

### qPCR

Total RNA was extracted by using TRizol, followed by cDNA synthesis by RevertAid First Strand cDNA Synthesis Kit (Thermo Scientific, USA), according to the manufacturer’s guidelines. Relative gene expression of TUSC3 gene in HGF1 and CAL 27 cell lines were evaluated by qPCR. In this reaction, beta actin was used as a reference gene and HGF1 as control cells. The reaction was carried out in qPCR machine (Agilent technologies stratagene MX3000P). Reaction conditions are 95 °C for 10 min, and 40 cycles of 95 °C for 30 s, 53 °C for 1 min and 72 °C for 1 min.

### PCR for TUSC3 deletion

TUSC3 gene deletion was detected by using a strategy of PCR to validate PCR reaction and internal control, beta globin was used. Sequence of both the genes was retrieved from online database i.e., Ensembl (https://asia.ensembl.org/index.html). Exon specific primer designing of conserved regions was done by using Primer3 software (http://primer3.ut.ee/). Primers for TUSC3 *(TUSC3 F*: ***5’****CCTCTGGAAAACCACTTGGA****3’***, *TUSC3 R*: ***5’****CGAACAGTTTTCTCTATGCAAGG****3’***) that gives *400 bp* product and beta globin (BG F: **5’**CAACTTCATCCACGTTCACC**3’**, BG R: **3’**GAAGAGCCAAGGACAGGTAC**5’**) which gives *268 bp* product, were commercially prepared by MOLEQULE-ON, New Zealand. Primers were reconstituted in 100 µM concentrations for PCR. Multiple concentrations and conditions were used during optimization of both genes in single reaction. The optimal concentration of reagents was; 4 µl of 50 ng/µl of genomic DNA, 12.5 µl of 2X Master Mix (MOLEQULE-ON, New Zealand), 0.25 µl of each 0.2 µM primers (i.e., *TUSC3 F, TUSC3 R, BG F* and *BG R)* and 7.5 µl of Milli Q water for volume make up to 25 µl per reaction. The reaction was followed by PCR steps which include first denaturation for 10 min at 94 °C, 35 cycles (94 °C for 30 s, 62 °C for 30 s and 72 °C for 30 s) and final extension at 72 °C for 7 min. Amplification products were run at 1.5% of agarose gel. Research grade agarose (MOLEQULE-ON, New Zealand) was dissolved in 100 ml of 1 X TBE buffer. A 5 µl of amplified product was loaded in the well and run at 100 V for 45 min. Gel documentation system (FastGene® FAS V, nippon genetics Germany) was used to analyze results of electrophoresis.

### ARMS PCR for p21 (*rs1801270*) Genotyping

**Fig. 1:**
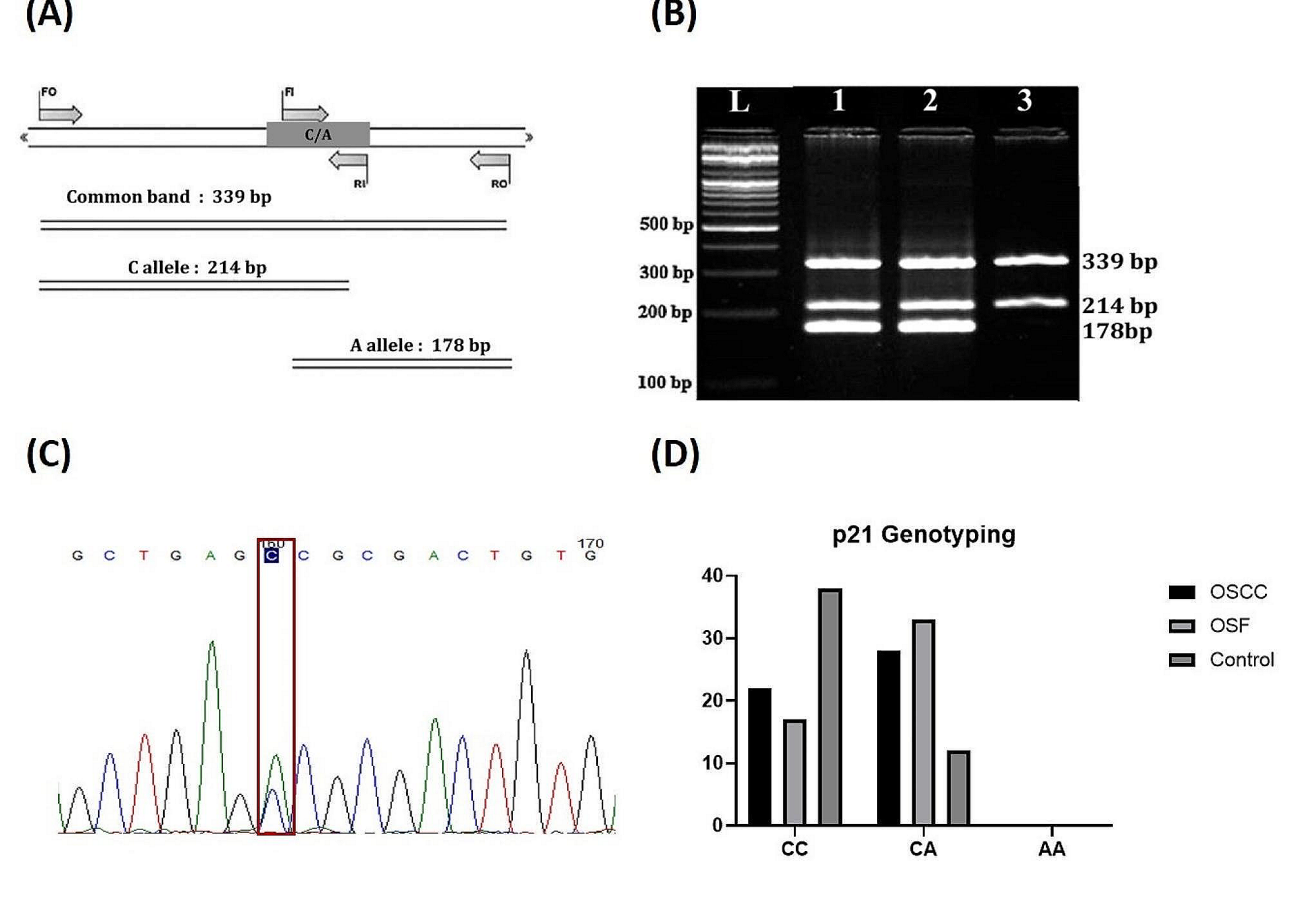
Genotyping of p21 *rs1801270* in OSF and OSCC Samples; (**A**) Schematic diagram of T-ARMS PCR product explanation for rs1801270. (**B**) Representation of band visualization of T-ARMS PCR products of rs1801270 on agarose gel. (**C**) Electrophorogram representing the rs1801270 polymorphism. (**D**) Genotypic frequency of homozygous C/C and heterozygous C/A in OSF and OSCC samples compared with controls

Genotyping of *rs1801270* was carried out through tetra primers amplification refractory mutation system (T-ARMS) PCR. For this, two pairs of primers were designed in which one pair was simple primers against targeted region and other pair was allele specific primer i.e., **C** and **G** (Fig. 1A). The sequences of common primers are; outer forward; *5*′*-GTCACCTGAGGTGACACAGCAAAGCCCGG-3*′ and outer reverse; *5*′-*GCCCCGTGGGAAGGTAGAGCTTGGGCAG-3*′, while sequences for allele specific primers are; inner forward: *5*′*-GCCCAGTGGACAGCGAGCAGCTGGGA-3*′ and inner reverse: *5*′*-CCCGCCATTAGCGCATCACAGTCGTGG-3*′.

### PCR for p53 amplification

Conventional PCR was used to amplify exon 4 of p53. A set of exon specific primers was designed; exon 4 forward primer 5’-TGCTCTTTTCACCCATCTAC-3’ and exon 4 reverse primer 5’-ATACGGCCAGGCATTGAAGT-3’. An optimized concentration and volume of reagents were used to prepare 50 µl of reaction (200 ng of gDNA, 5 µl of 10X PCR buffer, 4 µl of 2.5 mM dNTP mix, 0.5 µl of 20 µM of each primer, 0.2 µl of 5U of Taq polymerase, and 5.8 µl of nuclease free water), followed by thermal cycles at; first denaturation at 94 °C for 4 min, 30 cycles (denaturation 94 °C for 30 s, annealing at 61 °C for 35 s and extension at 72 °C for 30 s), second hold at 72 °C for 7 min, and storage at 4 °C until next step.

### DNA sequencing

Amplified products of p53 exon 4 and p21 were purified and sent for commercial Sanger sequencing (MoleQule-ON, Auckland, Newzeland).

### Data analysis

The resulting data were statistically analyzed with Chi square χ^2^ tests in order to investigate association between demographics and lesions studied, using, GraphPad Prism version 8.0 and odds ratio by an online free available software MedCalc. (https://www.medcalc.org/calc/odds_ratio.php). The sequence data was analyzed by Molecular Evolutionary Genetics Analysis Version 7.0 (MEGA 7) was used. The *p* value < 0.05 was considered as significant.

## Results

### Demographics

#### Age distribution

**Fig. 2:**
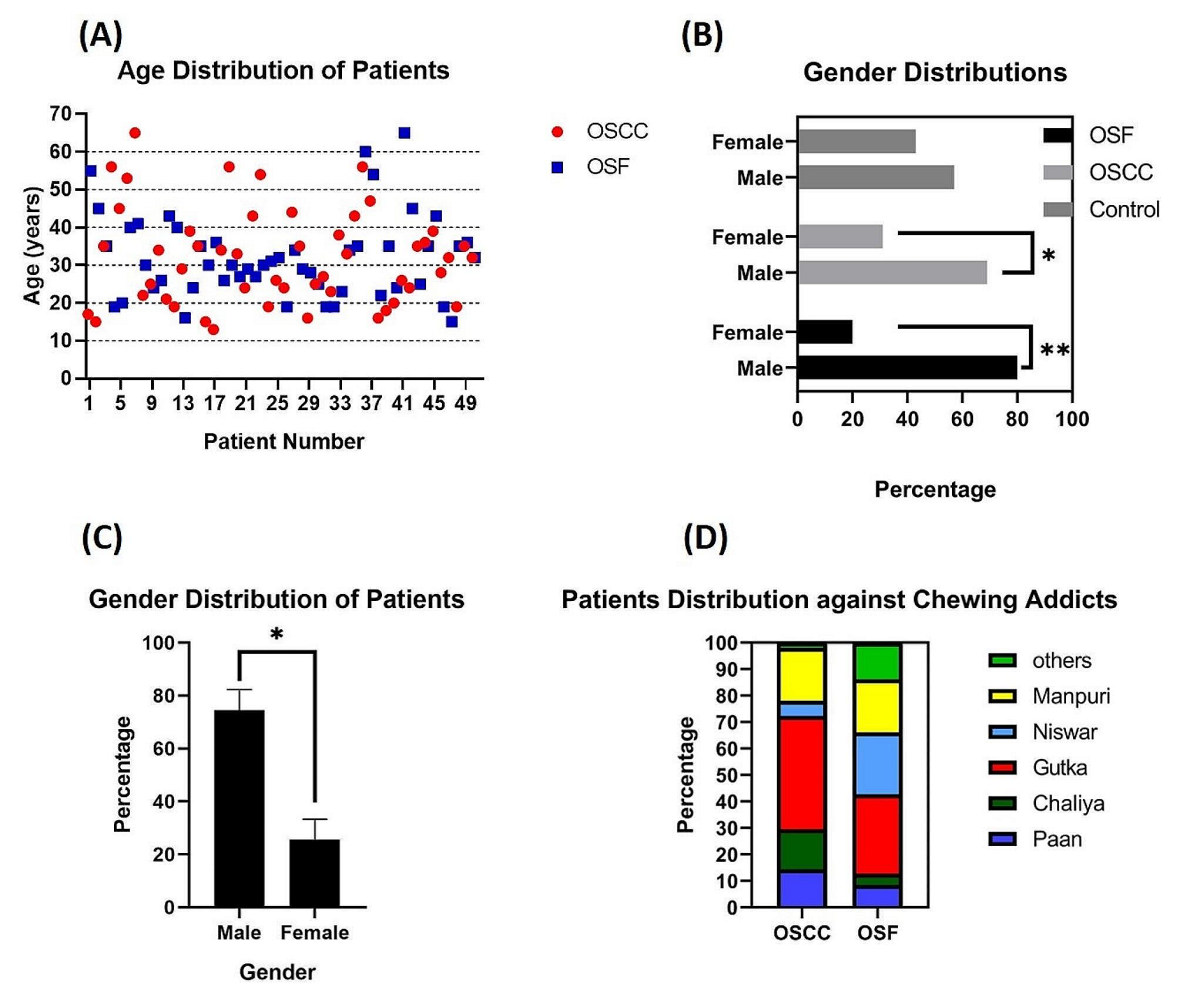
Demographics: (**A**) Age Distribution of recruited participants. (**B**) Gender Distribution of recruited participants according to condition type. (**C**) Overall gender distribution of recruited participants. (**D**) Distribution of recruited participants according to their chewing addictions. *: *P* < 0.05; **: *P* < 0.01

The age of all patients (OSCC and OSF) was found extensively distributed between 13 and 65 years with mean of 31.96 years. The cluster of patients was observed between 20 and 40 years of age (Fig. 2A).

#### Gender distribution

When OSF and OSCC patients were distributed according to gender, it was found that both the conditions were more frequent in males as compared to females (Fig. 2B). Overall Male-female ratio among recruited patients was almost 2.5:1 (Fig. 2C).

#### Addicts distribution

Patients were distributed according to their chewing habits. It showed that in both conditions i.e., OSF and OSCC, the percentages of *gutka* users were very high (42.8 and 30 ) followed by manpuri (20 and 20), Niswar (5.7 and 23.4), Chaliya (15.1 and 4.2), paan (14.4 and 8.4) and others (2 and 13.8) (Fig. 2D).

### Expression analysis of TUSC3 gene in cell culture settings

**Fig. 3:**
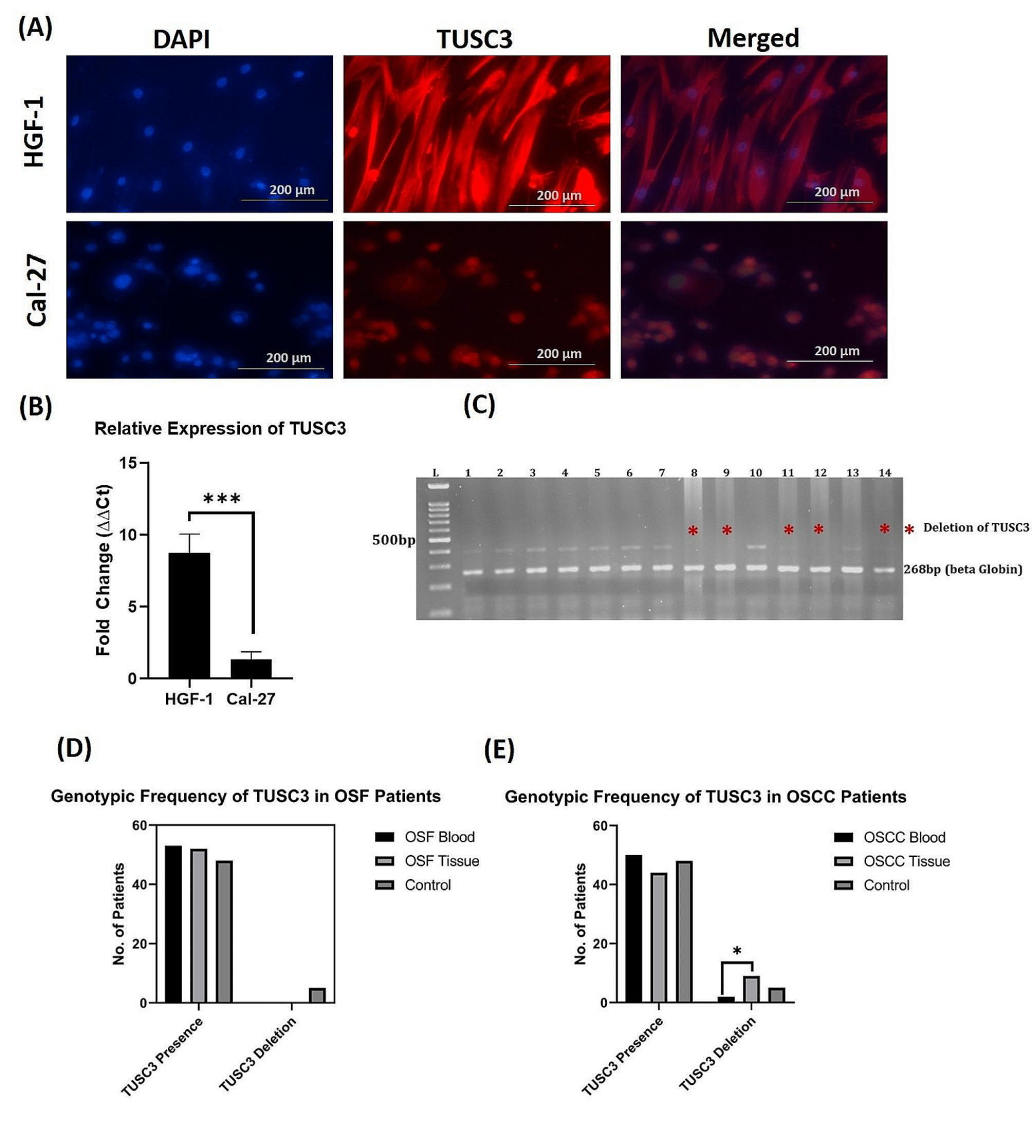
Analyses of TUSC3 expression in Cells and genetic deletion in clinically confirmed cases;(**A**) Immunofluorescence images of HGF-1 cells and Cal27 cells for TUSC3 expression. (**B**) Relative expression of TUSC3 in HGF-1 cells and Cal27 cells. (**C**) Bands representation of PCR product for TUSC3 and beta globin for deletion analysis on agarose gel. (**D**) Genotypic frequency of TUSC3 in OSF Samples. (**E**) Genotypic frequency of TUSC3 in OSCC Samples. Bars represent Standard deviation of data; *: *P* < 0.05; ***: *P* < 0.001

We analyzed the expression of TUSC3 in established cell lines of oral cancer (Cal-27) against normal cells (HGF-1), to check whether this gene has some different pattern of expression in both conditions or not. We found a significant evident that the expression of TUSC3 was significantly decreased in cancerous conditions at protein and transcript level which indicates the deletion of TUSC3 deletion (Fig. 3A & B). These results support our hypothesis that in clinical patients of OSF and OSCC might have deletion of TUSC3.

### Analysis of TUSC3 deletion in OSF samples

#### Frequency distribution

Genotyping of TUSC3 was performed by using multiplex PCR. Under optimized conditions with specific primers for TUSC3 and beta globin, it gives two products of 400 bp and 268 bp, respectively, while deletion of TUSC3 showed single band of 268 bp i.e., the band of internal control (Fig. 3C). The genotyping of TUSC3 was carried out in blood and tissue samples of 50 individuals who were clinically diagnosed as OSF patients along with 50 healthy individuals i.e., controls. Genotyping was performed to check the presence or deletion of TUSC3 in respective samples. We did not find deletion of TUSC3 in any clinical samples of OSF neither in blood nor tissues. Interestingly, 10% of all controls showed deletion of TUSC3 in their blood (Fig. 3D).

#### Association of TUSC3 with OSF

To explore the association of TUSC3 with OSF development, we performed Chi square and Odds ratio analyses. Acquired results showed that there was no significant association of TUSC3 gene deletion in OSF cases. Likewise, the Odds ratio also confirmed that the deletion of TUSC3 gene is not associated with OSF in available cases (Table 1).

### Analysis of TUSC3 deletion in OSCC samples

#### Frequency distribution

TUSC3 deletion frequency was also evaluated in OSCC blood and tissue samples, and in normal blood. We found a different pattern of TUSC3 deletion. There was a frequent deletion in the tissue samples i.e., > 22%, while the frequency of deletion was 3.9% in blood samples and 10% in normal blood (Fig. 3E).

#### Association of TUSC3 with OSCC

The association of TUSC3 deletion with OSCC development was calculated through Chi square (χ^2^). It showed that deletion of TUSC3 in blood samples of OSCC patients when tested against controls has non-significant association. Blood samples of the patients tested against their own tissue samples, gave a Chi square value of 8.02 with *p* < 0.05 (Table 1). These results provide strong evidence that deletion of TUSC3 gene in tissues is associated with the development of OSCC in comparison to its deletion in blood.

The odds ratio (OR) also provides a significant association of TUSC3 deletion with OSCC prognosis. Table 1 shows that the patients with deletion of TUSC3 in their tissues have more than 7 times likelihood to develop OSCC as compared to those patients who have similar deletion in their blood. However, the OR showed a protective effect of TUSC3 gene deletion in blood against OSCC development when it compared with controls having deletion in blood.

### Analysis of TUSC3 deletion in Transformation of OSF into OSCC

The association analysis of TUSC3 deletion and the development of OSCC from OSF was carried out separately in blood and tissue samples. Chi square value of TUSC3 deletion in OSF blood was found non-significant to associate with the development of OSCC, while in tissue samples, the Chi square results provided strong evidence that TUSC3 deletion is significantly associated with OSCC (Table 1). The OR results supplemented the chi square results showing that individuals having OSF have more than 30 times likelihood to develop OSCC when they undergo deletion of TUSC3 in their OSF lesions. However, OR for deletion in blood showed 5 times increased chances to develop OSCC from OSF.

### Genotyping of p53 gene exon 4 in OSF and OSCC samples

**Fig. 4:**
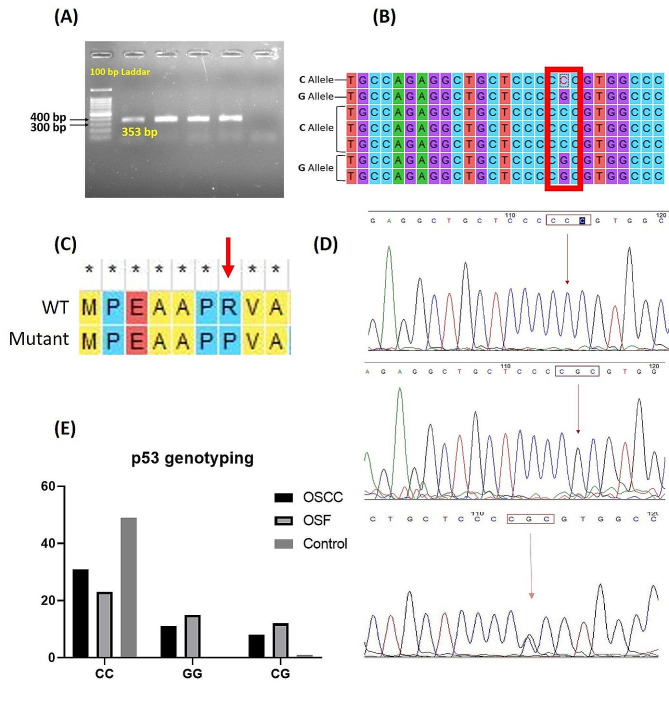
Genotyping of p53 gene exon 4 in OSF and OSCC Samples;(**A**) Representation of band visualization of PCR product for TP53 exon 4 products on agarose gel. (**B**) Multiple alignment of exon 4 of TP53 sequences representing change of C allele and G allele. (**C**) Translated protein sequence of exon 4 will G allele representing the change of arginine to proline. (**D**) Electrophorogram representing the polymorphism of exon 4 with homozygous C/C, heterozygous C/G, and homozygous G/G alleles. (**E**) Genotypic frequency of homozygous C/C, heterozygous C/G, and homozygous G/G in OSF and OSCC samples compared with controls

Exon 4 of p53 was amplified and visualized on agarose gel; a band at 353 bp confirms its amplification (Fig. 4A). The band was extracted and purified followed by sequencing. Analyses of sequenced amplicons on MEGA 7 revealed a single nucleotide change of **C** to **G** at position 119 of exon 4 of p53 gene (Fig. 4B). At codon 72, this polymorphism changes an amino acid proline to arginine (Fig. 4C). This polymorphism is represented by three genotypes; homozygous C/C, heterozygous C/G, and homozygous G/G (Fig. 4D). Among all three alleles, the genotypic frequency of CC was found to be frequent in OSCC and OSF tissues. The homozygous GG and heterozygous CG have comparatively equal ratio of occurrence (Fig. 4E). In OSF patients, the frequency of substitutive alleles (GG and CG) for proline to arginine change was found higher than OSCC patients. The relation of developing OSF and OSCC in accordance with this polymorphism was found significant when tested statistically, chi square values showed strong association χ^2^ (2, *N* = 100) = 20.49, *p <* 0.0001 and χ^2^ (2, *N* = 100) = 33.70, *p <* 0.0001, respectively. Corresponding values of odds ratio supplemented the association developed by chi square.

### Genotyping of p21 *rs1801270* in OSF and OSCC samples

The association of p21 *rs1801270* with OSCC and OSF development was analyzed by T-ARMS PCR. Figure 1A explains the allelic distribution of samples on agarose gel, the 339 bp band shows the entire amplicon of two outer primers, whereas the bands at 214 bp and 178 bp show the amplicons of specific alleles (CC and CA) of allele specific primers. Lane 1 and 2 of Fig. 1B indicate heterozygous CA allelic situation of *rs1801270*, while lane 3 indicates homozygous CC allele. The products of T-ARMS PCR for *rs1801270* were further validated by Sanger sequencing (Fig. 1C). The genotypic frequency of heterozygous CA was found relatively higher than homozygous CC, in all samples of patients as compared to healthy individuals (Fig. 1D). The homozygous AA was not found in any of the patient’s sample. Chi square test and odds ratio revealed that p21 (*rs1801270*) significantly increases the pathogenesis of OSCC and OSF, {χ^2^ (1, *N* = 100) = 10.67, *p <* 0.001 and χ^2^ (1, *N* = 100) = 17.82, *p <* 0.0001} respectively and corresponding values of odds ratio authenticate the results (Table 1).

**Table 1:** Chi Square and odds ratio of TUSC3, TP53 and p21 alterations with OSCC and OSF

Gene	Condition	Scenario	Chi Square	*p* value	odds ratio	95% confidence Interval	
						**Upper limit**	**Lower limit**
TUSC3	OSF	OSF blood vs. control	5.263	0.028	0.0819	0.0044	1.5228
		OSF Tissue vs. OSF blood	na	NA	1	0.0195	51.38
	OSCC	OSCC blood vs. control	1.51	0.202	0.36	0.0665	1.9483
		OSCC Tissue vs. OSCC blood	8.02	< 0.05	7.3171	1.5486	34.5739
	OSCC vs. OSF	OSCC blood vs. OSF blood	1.962	0.257	5	0.2341	106.783
		OSCC tissue vs. OSF tissue	12.814	< 0.05	30.4217	1.7485	529.3138
p53	OSF		20.49	< 0.0001	30.0323	3.8254	235.7759
	OSCC		33.7	< 0.0001	57.52	7.3569	449.749
p21 (*rs1801270*)	OSF		17.82	< 0.0001	6.147	2.457	14.61
	OSCC		10.67	< 0.001	4.030	1.649	9.167

## Discussion

The study of TUSC3 gene in pre-cancerous and cancerous conditions is very limited. TUSC3 has been reported as a potential biomarker in renal cell carcinoma [22]. A study reported significantly decreased expression of this gene in acute lymphoblastic leukemia in children [23]. A study demonstrated that cancer stem cell like phenotype can be induced by targeting TUSC3 in glioblastoma through miR-132 [24]. The regulation of TUSC3 gene expression through epigenetics was considered as a critical factor in the progression of colon cancers at cellular level [25].

In recent years, TUSC3 is considered a potential target for the treatment and diagnosis of different cancers; e.g. cervical cancer treatment, hepatocellular carcinoma where its downregulation could be a good biomarker for diagnosis [26], colorectal cancer where methylation of TUSC3 is suggested as a potential marker in peripheral blood cells [27], as well as in non-small lung cancer and gastric cancer [28]. Thus, the role of TUSC3 is quite significant in the progression of many cancers. However, its role in the development of OSCC is still unclear. In this study, we explored its role in pre-cancerous and cancerous conditions.

First, we analyzed the expression of TUSC3 in the OSCC cell line, and found significant downregulation when compared with HGF-1 cells. This confirms that TUSC3 has a vital role in the development of OSCC. These results prompted us to explore more about the association of TUSC3 deletion with OSCC as well as OSF development in clinically confirmed cases.

Like OSCC, TUSC3 studies with reference to OSF are very limited. However, studies on other genes have highlighted alterations in genetic mechanisms that may initiate excessive deposition of collagen in OSF. Studies on matrix metalloprotease, TGF-β, GSTM1, HIF‐1α, CYP1AI, CYP2E1, GSTT1, p14, p15 and p16 suggested their association with the susceptibility of OSF [29–31]. One study reported that TUSC3 was downregulated in OSF [32], but our results of genotyping did not find any association of TUSC3 in the development of OSF in clinically confirmed cases. A study by Ribeiro et al., reported that the loss of TUSC3 was a significant indicator of malignancy in oral cells [33]. We found that patients with OSCC have significant association with TUSC3 deletion in their tissue when compared with their blood samples and controls. This indicates that this deletion is very crucial at the somatic level and have a strong association with excessive use of tobacco and related products.

The addictive use of areca nut, betel quid, tobacco and its derived products has been reported to cause cytotoxic and genotoxic effects [4]. In Pakistan, the use of areca nut is much higher as compared to other materials. The use of derivatives of areca nut *(gutka, manpuri and flavored chaliya)* is very consistent in this region [11]. The carcinogen found in areca nut is arecoline N-oxide which is reported as the major contributor of carcinogenesis of oral cavity [34]. Many studies have identified the molecular mechanism of arecoline N-oxide in the development of OSF and its transformation to OSCC. Some of these studies indicated that this carcinogen causes dysfunction in the tumor suppressor genes and ultimately disturbs the cell cycle by G1/S transition. As a result, cells with damaged DNA bypasses the stage of cell cycle arrest and start to grow [4].

## Conclusion

In conclusion, our study provides a crucial insight into genetic factors that underlying OSCC and OSF. Analysis revealed a significant association between TUSC3 deletion and OSCC development. Additionally, polymorphisms in TP53 and p21 were significantly associated with OSCC and OSF, contributing to their pathogenesis. Importantly, our findings highlight a strong correlation between TUSC3 deletion and the excessive use of tobacco and related products. In brief this study emphasized the importance of genetic alterations in OSCC development and the need for further research into molecular mechanisms for improved diagnosis and treatment.

### Electronic supplementary material

Below is the link to the electronic supplementary material.

Supplementary Material 1

Supplementary Material 2

Supplementary Material 3

## Data Availability

The datasets generated or analysed during the current study are available in the NCBI GenBank repository with accession PP845877-PP845878.

## References

[1] Bray F, Laversanne M, Sung H, Ferlay J, Siegel RL, Soerjomataram I, Jemal A. Global cancer statistics 2022: GLOBOCAN estimates of incidence and mortality worldwide for 36 cancers in 185 countries. CA Cancer J Clin. 2024. 10.3322/caac.21834 38572751

[2] Miranda-Filho A, Bray F (2020). Global patterns and trends in cancers of the lip, tongue and mouth. Oral Oncol.

[3] Nokovitch L, Maquet C, Crampon F, Taihi I, Roussel L-M, Obongo R (2023). Oral Cavity Squamous Cell Carcinoma Risk Factors: State Art.

[4] Senevirathna K, Pradeep R, Jayasinghe YA, Jayawickrama SM, Illeperuma R, Warnakulasuriya S, Jayasinghe RD. Carcinogenic effects of Areca Nut and its metabolites: a review of the experimental evidence. 2023;13(2):326–46. 10.3390/clinpract13020030 PMC10037666 36961055

[5] Shirzaiy M, Neshat FJJoRiD, Sciences M (2020). Effect of areca nut on oral health. Rev.

[6] Warnakulasuriya SJTOCP. Diagnosis, Management. Potentially malignant disorders of the oral cavity. 2020:141 – 58.

[7] Jian X, Jian Y, Wu X, Guo F, Hu Y, Gao X et al. Oral submucous fibrosis transforming into squamous cell carcinoma: a prospective study over 31 years in mainland China. 2021;25:2249–56. 10.1007/s00784-020-03541-9 32844258

[8] Meng L, Jiang Y, You J, Zhao P, Liu W, Zhao N et al. IRF4 as a novel target involved in malignant transformation of oral submucous fibrosis into oral squamous cell carcinoma. 2023;13(1):2775. 10.1038/s41598-023-29936-8 PMC9935854 36797470

[9] Hosein M, Mohiuddin S, Fatima NJJ (2015). Association between grading of oral submucous fibrosis with frequency and consumption of areca nut and its derivatives in a wide age group: a multi-centric cross sectional study from Karachi. Pakistan.

[10] Arain SS, Kazi TG, Afridi HI, Talpur FN, Kazi AG, Brahman KD et al. Estimation of nickel in different smokeless tobacco products and their impact on human health of oral cancer patients. 2015;67(7):1063–74. 10.1080/01635581.2015.1073758 26368676

[11] Saleem S, Azhar A, Hameed A, Khan MA, Abbasi ZA, Qureshi NR. Ajmal MJOo. P53 (Pro72Arg) polymorphism associated with the risk of oral squamous cell carcinoma in gutka, niswar and manpuri addicted patients of Pakistan. 2013;49(8):818 – 23. 10.1016/j.oraloncology.2013.04.004 23683469

[12] Jain AJSCC-H, Modalities T. Molecular pathogenesis of oral squamous cell carcinoma. 2019.

[13] Vo TTT, Wee Y, Cheng HC, Wu CZ, Chen YL, Tuan VP et al. Surfactin induces autophagy, apoptosis, and cell cycle arrest in human oral squamous cell carcinoma. 2023;29(2):528–41. 10.1111/odi.13950 34181793

[14] Yoshimura S, Kasamatsu A, Nakashima D, Iyoda M, Kasama H, Saito T et al. UBE2S associated with OSCC proliferation by promotion of P21 degradation via the ubiquitin-proteasome system. 2017;485(4):820–5. 10.1016/j.bbrc.2017.02.138 28257844

[15] Zarate AM, Don J, Secchi D, Carrica A, Galindez Costa F, Panico R et al. Study of the TP53 codon 72 polymorphism in oral cancer and oral potentially malignant disorders in Argentine patients. 2017;39(5):1010428317699113. 10.1177/1010428317699113 28459200

[16] Ragos V, Mastronikolis NS, Tsiambas E, Baliou E, Mastronikolis SN, Tsoukalas N et al. p53 mutations in oral cavity carcinoma. 2018;23(6):1569–72. 30610778

[17] Shamloo B, Usluer S. p21 in Cancer Research. 2019;11(8):1178. 10.3390/cancers11081178 PMC6721478 31416295

[18] Vašíčková K, Horak P, Vaňhara PJC, Sciences ML. TUSC3: functional duality of a cancer gene. 2018;75(5):849–57. 10.1007/s00018-017-2660-4 PMC11105401 28929175

[19] Lin VC, Huang S-P, Ting H-J, Ma W-L, Yu C-C, Huang C-Y et al. Vitamin D receptor-binding site variants affect prostate cancer progression. 2017;8(43):74119. 10.18632/oncotarget.18271 PMC5650327 29088772

[20] Yu X, Zhang J, Zhong H, Liu F, Liang N, Wang Y et al. Decreased tumor suppressor candidate 3 predicts poor prognosis of patients with esophageal squamous cell carcinoma. 2016;13(12):963. 10.7150/ijms.16381 PMC5165690 27994502

[21] Yu X, Zhai C, Fan Y, Zhang J, Liang N, Liu F (2017). TUSC3: a novel tumour suppressor gene and its functional implications. J Cell Mol Med.

[22] Yan Y, Chen Z, Liao Y, Zhou JJOL. TUSC3 as a potential biomarker for prognosis in clear cell renal cell carcinoma. 2019;17(6):5073–9. 10.3892/ol.2019.10161 PMC6507427 31186719

[23] Khalid Z, Lafta FM, Al-Rekabi A-ANG. TUSC3 expression in Childhood Acute LymphoblasticLeukemia Patients in Baghdad, Iraq.

[24] Cheng ZX, Yin WB, Wang ZY (2017). MicroRNA-132 induces temozolomide resistance and promotes the formation of cancer stem cell phenotypes by targeting tumor suppressor candidate 3 in glioblastoma. Int J Mol Med.

[25] Taniue K, Hayashi T, Kamoshida Y, Kurimoto A, Takeda Y, Negishi L (2020). UHRF1-KAT7-mediated regulation of TUSC3 expression via histone methylation/acetylation is critical for the proliferation of colon cancer cells. Oncogene.

[26] Deng R, Lu X, Hong C, Cai R, Wang P, Xiong L et al. Downregulation of TUSC3 promotes EMT and hepatocellular carcinoma progression through LIPC/AKT axis. 2022;20(1):485. 10.1186/s12967-022-03690-3 PMC9590144 36274132

[27] Siri G, Mosallaei M, Ehtesham N, Rahimi H, Mazarei M, Sabet MN, Behroozi JJABR. TUSC3 methylation in peripheral blood cells as a biomarker for diagnosis of colorectal cancer. 2023;12(1):174. 10.4103/abr.abr_396_22 PMC10410437 37564442

[28] Han X, Li B, Zhang SJB. Pharmacotherapy. MIR503HG: A potential diagnostic and therapeutic target in human diseases. 2023;160:114314. 10.1016/j.biopha.2023.114314 36736276

[29] Chaudhuri SR, Mukherjee S, Paul RR, Haldar A, Chaudhuri KJG (2013). CYP1AI and CYP2E1 gene polymorphisms may increase susceptibility to oral Submucous fibrosis among betel quid chewers of. East India.

[30] Khan I, Kumar N, Pant I, Narra S, Kondaiah, PJPo. Activation of TGF-β pathway by areca nut constituents: a possible cause of oral submucous fibrosis. 2012;7(12):e51806. 10.1371/journal.pone.0051806 PMC3526649 23284772

[31] Takeshima M, Saitoh M, Kusano K, Nagayasu H, Kurashige Y, Malsantha M et al. High frequency of hypermethylation of p14, p15 and p16 in oral pre-cancerous lesions associated with betel‐quid chewing in Sri Lanka. 2008;37(8):475–9. 10.1111/j.1600-0714.2008.00644.x 18284544

[32] Huang Y-F, Hansen MF, Yang H-WJJDS. Determining the genetic signature of oral submucous fibrosis by the use of laser capture microdissection and a cDNA microarray. 2006;1(2):66–73.

[33] Ribeiro IP, Marques F, Caramelo F, Pereira J, Patrício M, Prazeres H et al. Genetic gains and losses in oral squamous cell carcinoma: impact on clinical management. 2014;37:29–39. 10.1007/s13402-013-0161-5 PMC13004429 24353162

[34] Ko AM-S, Tu H-P, Ko Y-CJC. Systematic Review of Roles of Arecoline and Arecoline N-Oxide in oral Cancer and strategies to Block Carcinogenesis. 2023;12(8):1208. 10.3390/cells12081208 PMC10137008 37190117

