# Supplementary figures and images for: TUSC3, p53 and p21 genetic association with development of oral submucous fibrosis and oral squamous cell carcinoma among addictive tobacco chewers of Pakistan
Source: BMC Oral Health. 2024 Jul 11;24:780. doi: 10.1186/s12903-024-04501-5 (PMC11241966; doi:10.1186/s12903-024-04501-5)

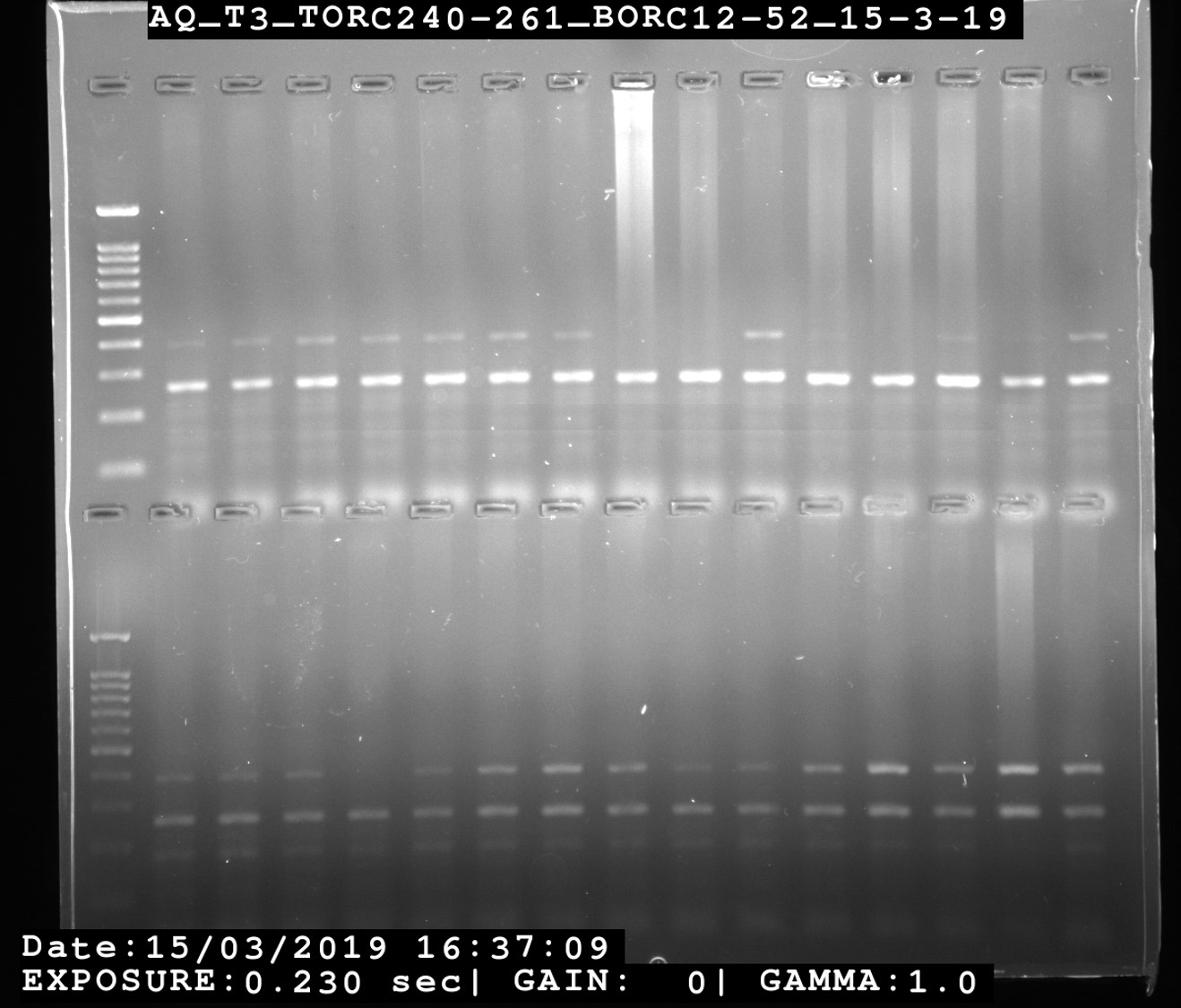

Supplement: Supplementary file 1 — Supplementary Material 1 [file 12903_2024_4501_MOESM1_ESM.jpeg]

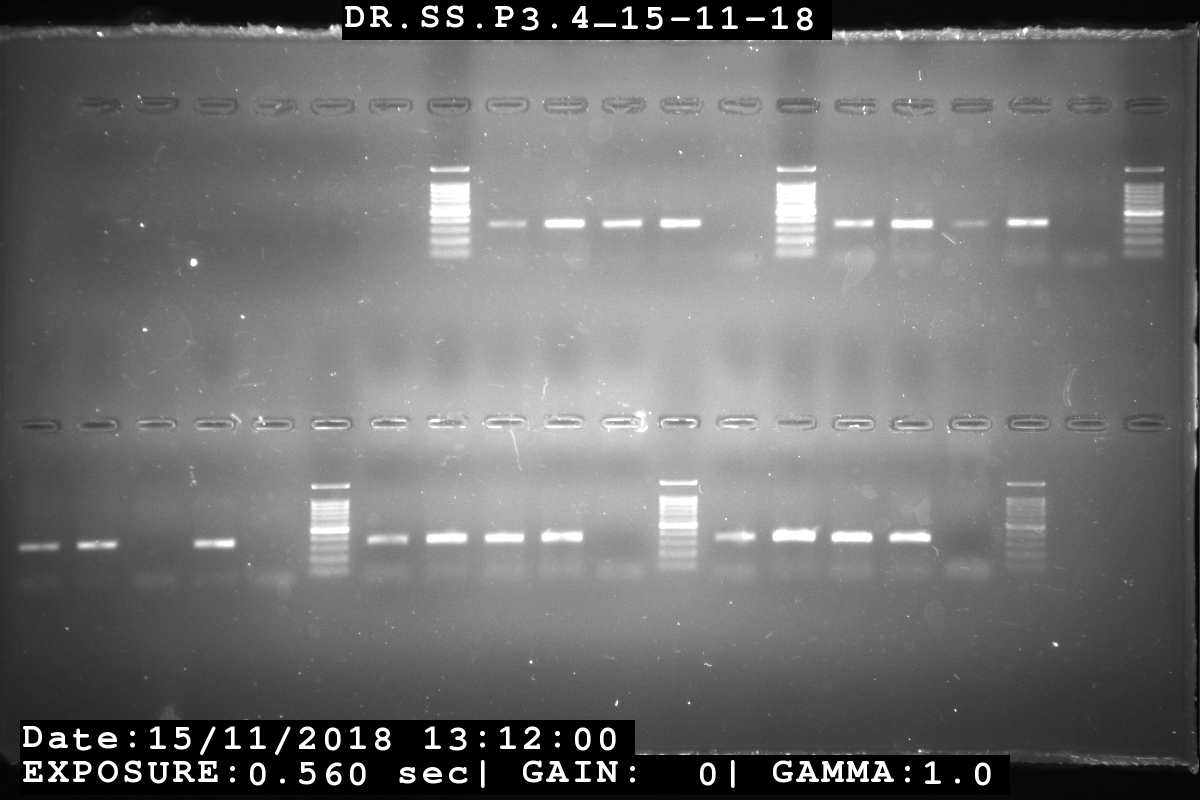

Supplement: Supplementary file 2 — Supplementary Material 2 [file 12903_2024_4501_MOESM2_ESM.jpeg]

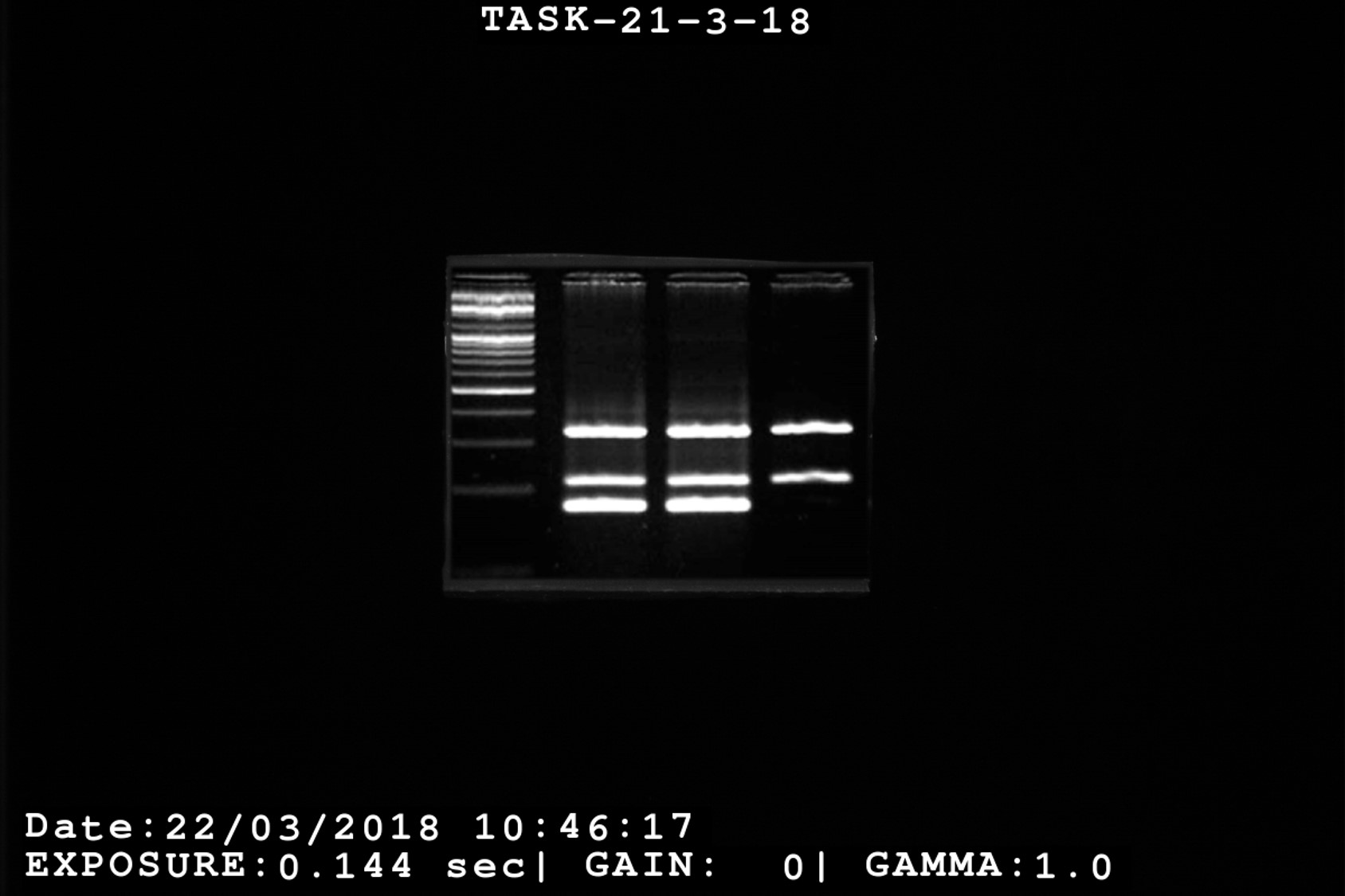

Supplement: Supplementary file 3 — Supplementary Material 3 [file 12903_2024_4501_MOESM3_ESM.jpeg]
